# Clinical and Molecular Spectrum of MYH9-Thrombocytopenia: Insights from a Single Centric Pediatric Cohort

**DOI:** 10.3390/children12111563

**Published:** 2025-11-17

**Authors:** Radu Obrisca, Andreea Serbanica, Andra Marcu, Ana Bica, Cristina Jercan, Irina Avramescu, Letita Radu, Cerasela Jardan, Anca Colita

**Affiliations:** 1 Department 7—Pediatrics, Faculty of Medicine, Carol Davila University of Medicine and Pharmacy, 020021 Bucharest, Romania; andreea.serbanica@umfcd.ro (A.S.); andra.marcu@umfcd.ro (A.M.); ana-maria.birsan@drd.umfcd.ro (A.B.); cristina.jercan@umfcd.ro (C.J.); irina.avramescu@drd.umfcd.ro (I.A.); letitia.radu@umfcd.ro (L.R.); cerasela.jardan@umfcd.ro (C.J.); anca.colita@umfcd.ro (A.C.); 2 Fundeni Clinical Institute, 020021 Bucharest, Romania

**Keywords:** thrombocytopenia, MYH9, congenital, NGS

## Abstract

**Background:** MYH9-related disease (MYH9-RD) is the most common form of inherited thrombocytopenia (IT). It is caused by pathogenic variants in the MYH9 gene. It manifests as early-onset macrothrombocytopenia with variable later-onset extra-hematological features, including hearing loss, renal disease, and cataracts. In pediatric patients, early recognition is critical to avoid misdiagnosis as immune thrombocytopenia (ITP) and unnecessary immunosuppressive therapy. **Methods:** We conducted a retrospective unicentric study at the Pediatric Oncology and Hematology Department, Fundeni Clinical Institute, Bucharest, Romania, including patients aged 0–18 years with suspected IT, tested between 2017 and 2025 by next-generation sequencing (NGS). Clinical, laboratory, and genetic data were reviewed. **Results:** Among 66 patients who underwent genetic testing, 31 (48.5%) had IT-associated genetic variants; 8 (25.8%) carried MYH9 mutations. Four patients (50%) had disease onset before age 1 year, three with neonatal presentation; 3 (37.5%) reported a family history of thrombocytopenia. Six variants were previously reported, and two were novel variants. Five variants (62.5%) were pathogenic, while three (37.5%) were initially classified as variants of uncertain significance (VUS). Most mutations were missense in the coiled-coil tail domain, correlating with milder thrombocytopenia and absence of extra-hematological features. No life-threatening bleeding was recorded; hemorrhagic symptoms were limited to minor mucocutaneous bleeding. **Conclusions:** This is the first Romanian pediatric cohort and one of the few existing pediatric cohorts describing the genetic and clinical spectrum of MYH9-RD. Early genetic confirmation enables precise diagnosis, tailored management, and family screening, while preventing inappropriate therapies.

## 1. Introduction

Immune thrombocytopenia (ITP) remains the most common form of childhood bleeding disorder in the presence of isolated thrombocytopenia [1]. However, due to the fact that the diagnosis remains one of exclusion, up to 15% of patients are misdiagnosed at some point during disease evolution [2]. Due to the rapid evolution of genetic and molecular biology diagnostic tools, an increasing number of gene mutations have been found to cause different forms of inherited thrombocytopenia (IT) [3]. Despite the fact that these disorders are often associated with other phenotypical anomalies, isolated thrombocytopenia is, in some cases, the first and only manifestation of disease.

MYH9-related disease (MYH9-RD) is an autosomal dominant genetic disorder and is the most common form of IT [3,4]. MYH9 is a large gene localized on chromosome 22q12.3. It contains 41 exons and encodes the heavy chain of non-muscle myosin of class II, isoform A [5,6]. It encompasses four dominant disorders, which, until its discovery in 1999, were thought to be distinct pathologies: May–Hegglin anomaly, Epstein Syndrome, Fechtner syndrome, Sebastian platelet syndrome, and autosomal dominant deafness DFNA17 [4,5,6,7,8]. Mutations in the MYH9 gene lead to premature release of macro-thrombocytes from the bone marrow and neutrophils with pale blue inclusions bodies, which are NMHC IIA and are called Dohle inclusions [5,6,8]. About 80–90% of patients present missense mutations, with nonsense and splice-region mutations having a lower incidence. Other mutation types, like small deletions or insertions, are rarely reported [5,7]. Mutations in the head domain (exons 1–19), which are less common than the tail domain, have the most severe phenotypical manifestations, with severe thrombocytopenia as well as poorer platelet function and early onset of extra-hematological manifestations [9]. Mutation of the helical tail domain (exons 20–39), which is the most commonly mutated region overall, tends to have milder thrombocytopenia, with no or later onset of systemic phenotypical manifestation [8,9]. Non-helical tail mutations are rare; they associate with mild thrombocytopenia, with late onset, and rare systemic involvement [8]. All affected patients develop thrombocytopenia, which can remain the only sign of disease throughout life [6,7].

Thrombocytopenia is frequently the first sign of disease, with a degree that can range from mild platelet count decrease to severe thrombocytopenia, with platelet count being underestimated when using an automated cell counter, in the case of severe macrocytosis [9,10]. Severe bleeding episodes are scares, with most patients presenting only with minor bruising. Therapeutic challenges present when facing procedures with a high blending risk, such as surgery [8,9].

Other manifestation usually occurs during adulthood. Deafness is the second most frequent disease manifestation, with features of progressive bilateral sensorineural hearing loss, occurring most frequently after 30 years old, and affecting most patients over time [9,11]. Early onset, in adolescence or young adulthood, leads to severe, profound deafness [5].

A quarter of patients will develop kidney damage, which presents as proteinuric nephropathy. Disease onset occurs before 35 years old, and has an aggressive evolution towards end-stage kidney disease with the requirement of dialysis or renal transplant [9,12].

Other manifestations consist of presenile bilateral cataract, which occurs in 20% of patients, and persistent, chronic elevation of liver enzymes, but with no liver dysfunction associated [13,14].

Diagnosis is suspected in patients with macro-thrombocytopenia, especially in the presence of giant platelets, frequently larger than erythrocytes, or other clinical manifestations, but it usually occurs in adult patients [5]. Confirmation of diagnosis can be performed through genetic testing or by identifying the Dohle-like inclusions in neutrophils using immunofluorescence.

## 2. Materials and Methods

We performed a retrospective unicentric study that included patients who underwent genetic testing for IT from the Pediatric Oncology and Hematology Department of the Fundeni Clinical Institute, Bucharest, Romania, between 2017 and 2025. The primary objective was to assess the incidence, clinical and paraclinical aspects of MYH9-RD, as well as individual variant characterization. The study was conducted in accordance with the Declaration of Helsinki, and the protocol was approved by the Ethics Committee, registration number 56,258 on the 18th of October 2022.

Inclusion criteria were patients with IT suspicion, with a genetic test performed using next-generation sequencing (NGS), aged 0 to 18 years old, at the moment of initial presentation, with signed informed consent by a legal representative. NGS was performed on a peripheral blood sample using either gene panels or whole exome sequencing in off-site laboratories. Thrombocytopenia was defined as a platelet count lower than 100 × 10^9^/L. Chronic thrombocytopenia was defined as thrombocytopenia with an evolution lasting more than 12 months. Refractory thrombocytopenia was defined as persistent thrombocytopenia that does not respond to first-line treatment. Patients who met at least one of the following criteria underwent genetic testing: age under 1 year at thrombocytopenia onset with IT suspicion; chronic/refractory thrombocytopenia; family history of thrombocytopenia. Variants initially classified as having uncertain significance (VUS) were reevaluated according to the updated ACMG/AMP 2024 criteria. The reclassification process was conducted by two geneticists and a clinical hematologist experienced in the diagnosis of inherited thrombocytopenia. The reevaluation integrated multiple categories of evidence, including population data (allele frequency in the gnomAD database), computational pathogenicity prediction algorithms (PolyPhen-2, SIFT, MutationTaster), family segregation analysis when such data were available, as well as a review of the published literature and corresponding ClinVar records. Any discrepancies between evaluators were resolved through consensus discussion. The presence of macro-thrombocytes was assessed only on the peripheral blood smear. In case of severe thrombocytopenia, the mean platelet volume (MPV) was not quantified by the analyzer. The WHO bleeding scale was used to assess bleeding grade.

## 3. Results

**Figure 1:**
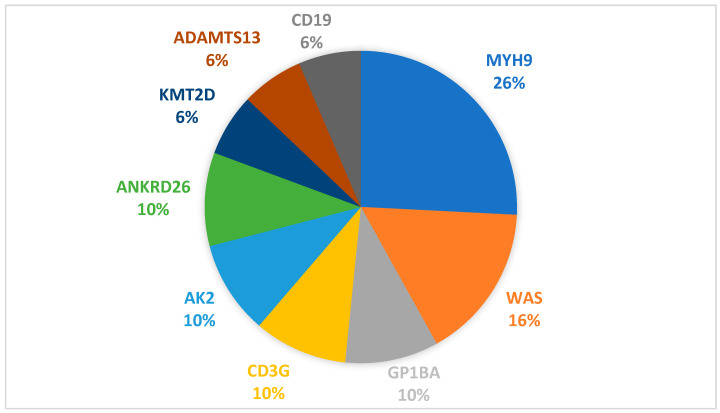
IT phenotype genetic variants distribution.

In total, 66 patients underwent genetic testing, with 31 patients (48.5%) associating genetic variants with phenotypic manifestations of IT. Of the 31 patients, 8 (25.8%) presented a mutation of the MYH9 gene (Figure 1). Patient characteristics are presented in Table 1. Four patients (50%) had disease onset before 1 year old, with 3 of them (37.5%) having neonatal debut. The median follow-up period is 32 months (range 6–60 months).

**Figure 2:**
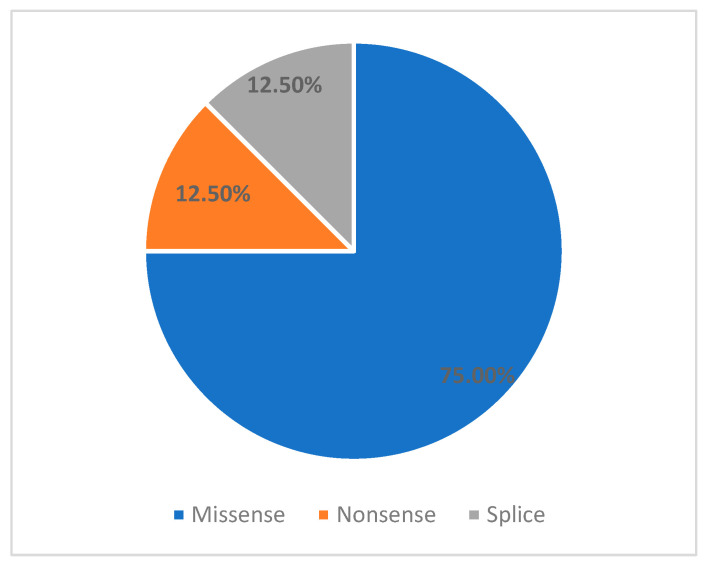
Distribution of mutation type.

**Figure 3:**
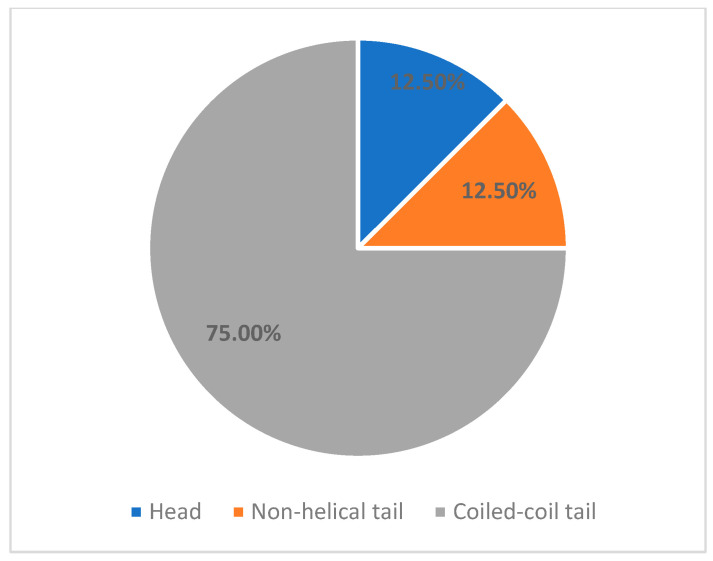
Incidence of mutation location.

We identified six variants that have been previously reported in the medical literature, as well as two novel variants. Of these variants, 5/8 (62.5%) were classified as pathogenic, and 3/8 (37.5%) were initially classified as variants of unknown significance (VUS) (Table 2). Most mutations were missense (Figure 2) and affected the coiled-coil tail domain (Figure 3). Following this comprehensive assessment, two of the three initially reported VUS variants were reclassified as Likely Pathogenic (Table 3).

## 4. Discussions

### 4.1. Variant Breakdown

1. MYH9 c.3493C>T pathogenic variant
  Patient: 14-year-old male, chronic thrombocytopenia for 7 years.
  Previous treatment: Short-course corticosteroid.
  Clinical and laboratory findings at first admission: Mild thrombocytopenia (60 × 10^9^/L) macro-thrombocytes on the peripheral blood smear. Mild bruising.
  Family history: Father, paternal grandfather, uncle.
  Disease evolution to date: 25 months; mean platelet count of 32 × 10^9^/L (range 10–60 × 10^9^/L). No life-threatening hemorrhagic events, maximum WHO bleeding scale grade 1. No extra-hematological involvement.
  Genetic aspects: Familial testing revealed that the patient’s father was also carrying the genetic variant. There are multiple entries in the ClinVar database that support the germline pathogenic classification of this genetic variant and association with phenotypical manifestations of disease featuring thrombocytopenia and deafness [15,16].
2. MYH9 c.4270G>A pathogenic variant—2 patients
  - Patient: 21-month-old female, refractory chronic thrombocytopenia since birth.
    Previous treatment: Short-course corticosteroid and immunoglobulin.
    Family history: Negative. After diagnosis, thrombocytopenia was identified in patient’s father.
    Clinical and laboratory findings at first admission: Platelet 35 × 10^9^/L; macro-thrombocytes on the peripheral blood smear. Minor bruising and petechia.
    Disease evolution to date: 8 months; mean platelet count 42 × 10^9^/L (range 19 to 85 × 10^9^/L). No life-threatening hemorrhagic events, maximum WHO bleeding scale grade 1. No extra-hematological involvement.
  - Patient: One-year-old male, chronic thrombocytopenia since birth.
    Previous treatment: Short-course corticosteroid.
    Clinical and laboratory findings at first admission: Platelet count 36 × 10^9^/L, and macro-thrombocytes on the peripheral blood smear. Minor bruising and petechia.
    Family history: Mother, grandmother, aunt.
    Disease evolution to date: 36 months. No life-threatening hemorrhagic events, maximum WHO bleeding scale grade 1. No extra-hematological involvement.
    Genetic aspects: This variant has been previously identified in a Japanese family with 14 affected individuals from 4 generations with only thrombocytopenia being reported [17]. In a large Italian study, which included 255 patients from 121 families, the variant was identified in 13 patients from 8 families, and showed a lower risk for other congenital defects compared to other variants. This variant has also been reported in an adult patient with hypertension and proteinuria. Patient had no history of bleeding episodes or other disease manifestations, but presented thrombocytopenia in childhood [18]. No severe hemorrhagic events have been reported or other manifestations of disease in either patient.
3. MYH9 c.287C>T pathogenic variant
  Patient: 2-month-old male with severe thrombocytopenia at 24 h after birth.
  Previous treatment: Multiple short-course corticosteroid, 4 courses of intravenous immunoglobulin.
  Clinical and laboratory findings at first admission: Platelet count of 221 × 10^9^/L, macro-thrombocytes on the peripheral blood smear, bruising, and diffused petechiae. We discontinued all treatment pending results of genetic testing. In evolution platelet count decreased in 2 weeks to 4 × 10^9^/L, and associated transient neutropenia (0.5 × 10^9^/L).
  Bone marrow aspirate, which revealed increased megakaryocytes, no blast cells, normal granulocyte series.
  Family history: Negative.
  Disease evolution to date: 12 months, severe thrombocytopenia with platelet count below 10 × 10^9^/L. No life-threatening hemorrhagic events, maximum WHO bleeding scale grade 1. No extra-hematological involvement.
  The variant was first reported in 2002, in two families, one of which was also associated with hearing loss and nephropathy [19].
4. MYH9 c.5797C>T pathogenic variant
  Patient: 6-year-old female, chronic mild thrombocytopenia discovered at 8 months old.
  Previous treatment: No.
  Clinical and laboratory findings at first admission: Platelet count 62 × 10^9^/L, and macro-thrombocytes on the peripheral blood smear. No signs of bleeding.
  Family history: positive, multiple relatives.
  Disease evolution to date: 4 years; mean platelet count 66 × 10^9^/L (range 56–71 × 10^9^/L). No life-threatening hemorrhagic events, maximum WHO bleeding scale grade 1. No extra-hematological involvement.
  Genetic aspects: One of the most frequent variants that has been reported in ClinVar in patients with mild thrombocytopenia but seems to not be associated with other phenotypical anomalies [20,21,22,23].
5. MYH9 c3485+6C>T VUS
  Patient: 4-year-old female with chronic refractory thrombocytopenia from 10 months old.
  Previous treatment: Short-course corticosteroids and intravenous immunoglobulin.
  Clinical and laboratory findings at first admission: Severe thrombocytopenia with no macro-thrombocytes. Bone marrow analysis was normal, with a slight increase in megakaryocyte number. Bruising and petechia.
  Family history: Negative.
  Disease evolution to date: Patients started treatment with Romiplostim, with initial good response, reaching transitory platelet counts of up to 500 × 10^9^/L for up to 10 weeks at a time, but no stable response was achieved. Genetic testing was performed after TPO-RA loss of response.
  Monitorization period is 6 years, with patient maintaining platelet count <10 × 10^9^/L.
  Genetic aspects: This variant has not been previously reported in the medical literature or in ClinVar database. After variant reevaluation, it was maintained as a VUS. No life-threatening hemorrhagic events, maximum WHO bleeding scale grade 1. No extra-hematological involvement.
6. MYH9 c.5411A>G
  Patient: 21-year-old female, chronic thrombocytopenia from 10 years old.
  Previous treatment: Short-course corticosteroid.
  Clinical and laboratory findings at first admission: Platelet count of 39 × 10^9^/L, no macro-thrombocytes described on the peripheral blood smear.
  Family history: Negative.
  Genetic aspects: The variant was predicted to be tolerated by most in silico tools utilized in genetic testing; however, patient maintains phenotypic manifestation of disease with chronic thrombocytopenia. Variant reevaluation classified it as Likely Pathogenic based on PM1, PM2, PM6, and PP2 criteria. The variant has not been previously reported in the medical literature or in the ClinVar database. No life-threatening hemorrhagic events, maximum WHO bleeding scale grade 1. No extra-hematological involvement.
7. MYH9 c.3838G>A, VUS
  Patient: 9-year-old female.
  Previous treatment: Long-term corticosteroid and intravenous immunoglobulin
  Disease evolution to date: Patient was referred to our unit for refractory thrombocytopenia after 3 months of continuous prednisone use. The following year, patient maintained severe thrombocytopenia but also encountered multiple severe infectious episodes, including opportunistic infections with Clostridium Difficile. Primary immunodeficiency was suspected, and the patient received monthly treatment with intravenous immunoglobulin. Following treatment, patient achieved and maintained normal platelet count, even after treatment stop. Currently, almost 4 years after debut, platelet count maintains normal, with no treatment, maximum WHO bleeding scale grade 1.
  Family history: Negative.
  Genetic aspects: Variant reevaluation classified it as Likely Pathogenic based on PS4_moderate, PM1, PM6, and PP2.
  Comments: Although this variant fulfilled ACMG criteria supporting a Likely Pathogenic classification (PS4_moderate, PM1, PM6, PP2), the absence of sustained thrombocytopenia raises the possibility that this variant may represent an incidental finding rather than a driver of disease in this patient. This case highlights an important limitation of ACMG-based variant interpretation, which integrates population, computational, and limited clinical data, but does not always account for incomplete penetrance, variable expressivity, or competing clinical diagnoses.
  Previous reports have described this variant in association with heterogeneous phenotypes, including primary immunodeficiency and steroid-resistant nephrotic syndrome, but without definitive evidence establishing causality for MYH9-related thrombocytopenia.
  Accordingly, we acknowledge that the patient’s clinical course may reflect that the c.3838G>A variant requires further evidence—including familial segregation, functional studies, or additional phenotypically concordant cases—before definitive disease attribution can be established.
  This case underscores the necessity for cautious interpretation of VUS and Likely Pathogenic variants when clinical findings diverge from expected disease phenotypes.
  Our cohort confirms that MYH9-related disease is the most common genetically confirmed inherited thrombocytopenia in children. In our series, **26% (8/31)** of patients carrying IT-associated variants had an MYH9 mutation. This incidence is comparable to published pediatric and mixed-age IT cohorts, where MYH9-RD ranges from **20 to 40% of inherited thrombocytopenia cases** (23% in the Italian registry, 22–33% in East Asian pediatric cohorts, and ~30% in large multigene IT sequencing series) [3,7,8].
  The molecular spectrum in our patients revealed predominance of missense variants in the coiled-coil tail domain (6/8 patients, 75%), consistent with known MYH9 genotype distribution. These patients exhibited a mild hematologic phenotype, preserved hemostatic stability, and no extra-hematological manifestations during follow-up. In contrast, the single head-domain variant (c.287C>T) discovered in a 2-month-old was associated with the most severe hematological phenotype, recapitulating existing genotype–severity models associating motor domain variants with early disease onset, severe thrombocytopenia, and higher risk of early systemic disease [5,7,8,9,11].
  Non-hematologic manifestations were absent in this cohort, as renal, auditory, and ophthalmologic complications typically emerge in adolescence or adulthood. This reinforces the importance of anticipatory multidisciplinary surveillance, even in asymptomatic children [11,12].
  A key finding of our study is that 87.5% of genetically confirmed MYH9-RD patients were initially misdiagnosed and treated ITP, consistent with previously reported misdiagnosis rates of inherited platelet disorders [2,3] The consequences were substantial: unnecessary exposure to corticosteroids and IVIG in 7 of 8 patients, delayed molecular diagnosis (median 24 months; up to 11 years), Escalation to second-line therapies including thrombopoietin receptor agonists or repeated immunosuppression in some cases.
  These findings highlight a persistent diagnostic gap: macrothrombocytopenia alone remains insufficiently recognized as a red flag, despite being one of the strongest clinical discriminators against ITP [10,24]. Our results strongly support early NGS-based evaluation in infants, refractory or chronic cases, and patients with macrothrombocytopenia, aligning with emerging international recommendations advocating early genetic testing to prevent unnecessary exposure to immunosuppression, early monitorization plan for extra-hematological manifestation [3,25].

### 4.2. Treatment

A multidisciplinary approach is required for the proper management of patients with MYH9-RD, because the aim is not only to manage or prevent hemorrhagic episodes, but also to address the extra-hematological manifestations of the disease. Treatment options for patients with MYH9-related thrombocytopenia can be grouped into two categories: curative and hemorrhage prevention and management. In most cases, patients do not present with life-threatening bleeding, and hemorrhagic manifestation of disease is limited to cutaneous bleeding, so from a hematological standpoint, our patients are monitored with CBC and physical examination every 6 months [25].

The only currently available curative treatment for patients with MYH9-related thrombocytopenia remains hematopoietic stem cell transplant. However, due to significant transplant-related morbidity and mortality, as well as a mild hemorrhagic profile of disease, it is rarely used for these patients [26,27]. Due to the fact that in our cohort, the bleeding phenotype is mild, hematopoietic stem cell transplantation was not considered as a treatment option for any patient.

The goal for our patients is not to achieve a normal or improved platelet count. The main focus is to prevent and treat active bleeding episodes as well as monitor for early signs of extra-hematological manifestation.

Patients are instructed to avoid drugs that affect hemostasis (impair platelet function, decrease platelet count) as well as nephrotoxic drugs, such as non-steroidal anti-inflammatory drugs, some antibiotics, and oncological drugs [24,28,29,30].

The most feared complication remains peri-procedural excessive bleeding, with studies indicating that 15% of patients with MYH9-RD develop excessive bleeding during surgery [31]. Platelet transfusion remains the most effective treatment for managing active bleeding, or for pre-procedural transit increase in platelet count [25,28]. According to current guidelines, we recommend a minimum of 50 × 10^9^/L platelets for major surgery, and 100 × 10^9^/L for critical sites using ABO-matched, irradiated, preferably apheresis from a single donor, in order to reduce the incidence of transfusion-related complications [24,32,33,34]. We do not support the use of prophylactic platelet transfusions in the absence of clear bleeding risk situations.

In recent years, more and more studies have shown that MYH9-RD patients respond to thrombopoietin receptor agonists. The efficacy and safety of Eltrombopag was studied in patients with MYH9-RD with transient platelet response in up to 90% of patients [35,36,37]. The temporary improvement of platelet count was observed in our patient with the c3485+6C>T variant, who received TPO-RA before genetic testing. However, current indications limit the use of TPO-RA to off-label prescription in Romania.

Besides hematological monitoring, it is recommended for patients to undergo frequent nephrology, ENT, and ophthalmologic evaluation, especially in older patients. Proteinuria is one of the best markers for kidney damage, and should be monitored every 6–12 months [28]. Ophthalmologic evaluation as well as hearing test are recommended every 3–5 years [38]. If a cataract occurs, patients benefit from standard surgical procedures [28].

Early recognition is essential, as MYH9-RD has a distinct natural history, prognosis, and management strategy, and unnecessary exposure to treatment can be avoided with timely molecular investigation. Our data confirm the diagnostic relevance of NGS in pediatric thrombocytopenia, especially in patients presenting with early-onset disease, refractory or chronic thrombocytopenia, macro-thrombocytes, or positive family history [3,4,7]. One of the key findings of our study, which supports early implementation of molecular diagnosis, is the infant onset of disease present in half of the patients, with 37.5% presenting phenotypical manifestation in the first days of life.

Consistent with previous MYH9-RD cohorts, the phenotype observed in our patients was predominantly hematological, with mild-to-moderate thrombocytopenia and macro-thrombocytes, without any documented life-threatening hemorrhagic events [5,6,8]. None of our patients exhibited extra-hematological manifestations in accordance with prior genotype–phenotype studies that describe extra-hematological features as progressive, age-dependent complications typically emerging in adolescence or adulthood [9,10,11].

The strengths of this study include detailed variant-level characterization, integration of clinical and molecular review, and one of the few cohorts of pediatric MYH9-RD patients expanding genetic epidemiology data.

### 4.3. Study Limitations

The small cohort size limits statistical power, restricts genotype–phenotype correlation strength, and may not fully represent the clinical and mutational spectrum of pediatric MYH9-related disease. However, given the rarity of the disease, we acknowledge that this is one of the few existing MYH9-RD pediatric studies.

The retrospective, single-center design introduces the potential for selection and referral bias, as patients undergoing genetic testing were pre-selected based on clinical suspicion, chronicity, or treatment refractoriness, rather than enrolled systematically.

Although three VUS variants were reevaluated and reclassified, no functional validation studies (e.g., RNA splicing assays, protein modeling, or cellular expression studies) were performed to confirm pathogenic effects, particularly for the two novel variants, leaving residual uncertainty regarding causality.

Finally, the c.3838G>A case remains clinically unresolved, as the patient demonstrated phenotypic discordance, raising the possibility that the variant may be incidental rather than disease-causing. This case underscores the limitations of variant classification frameworks when molecular assignments lack strong clinical concordance.

## 5. Conclusions

This study represents the first comprehensive description of MYH9-related disease (MYH9-RD) in a Romanian pediatric cohort, providing novel insights into its clinical presentation, genetic spectrum, and diagnostic challenges. Our findings demonstrate that a significant proportion of children who present with thrombocytopenia harbor genetic variants associated with congenital disease, with MYH9 being the most frequently affected gene. Genotype–phenotype correlation of MYH9-RD was reflected in the generally mild hematological phenotype and absence of extra-hematological manifestations at the time of pediatric evaluation.

Importantly, more than two-thirds of patients had been initially misclassified and inappropriately managed as immune thrombocytopenia, highlighting the persistent gap in recognition of inherited platelet disorders. Early genetic testing proved essential for establishing the correct diagnosis, guiding monitoring strategies, and informing family screening.

The identification of two novel MYH9 variants adds to the mutational spectrum of this condition and underlines the ongoing need for variant reclassification in the light of emerging evidence. Given that MYH9-RD is a lifelong condition with potential for late-onset systemic complications such as nephropathy, hearing loss, and cataract, a multidisciplinary follow-up plan is critical, even in asymptomatic pediatric patients.

Our results reinforce the value of integrating molecular diagnostics into the routine evaluation of pediatric thrombocytopenia and support international recommendations advocating for targeted NGS panels in suspected inherited platelet disorders. This approach not only avoids unnecessary exposure to immunosuppressive agents but also enables timely preventive measures and personalized patient management.

## Figures and Tables

**Table 1:** Patient characteristics.

Patients No.	8
Sex	
F	4 (50%)
M	4 (50%)
Age at first presentation	1.5 year
Median (range)	(0–14)
Platelet count—first presentation	35.5 × 10^9^/L
Median (range)	(7–126)
No. patients treated as ITP	7 (87.5%)
Disease time evolution until genetic confirmation	24 months
Median (range)	(2–132)
No. patients with prior family history	3 (37.5%)

**Table 2:** Genetic variant characterization.

Variant	Location	Exon	Type	Classification	Reclasification	Age at Onset	Family History	Thrombocyte Levels (×10^9^/L)	Macro Thrombocytopenia
c.3494C>T	Coiled-coil Tail	27	Missense	Pathogenic	N/A	7	Yes	50–60	Yes
c.4270G>A	Coiled-coil Tail	31	Missense	Pathogenic	N/A	At birth	No	40–85	Yes
c.4270G>A	Coiled-coil Tail	31	Missense	Pathogenic	N/A	At birth	Yes	40–50	Yes
c.287C>T	Head	2	Missense	Pathogenic	N/A	At birth.	No	<10	Yes
c.5797C>T	Non-helical Tail	41	Nonsense	Pathogenic	N/A	8 m.o.	Yes	60–70	Yes
c.3838G>A	Coiled-coil Tail	27	Missense	VUS/Benign	Likely Pathogenic	7	No	Normal	No
c3485+6C>T	Coiled-coil Tail	26	Splice-region	VUS	VUS	10 m.o.	No	<10	No
c.5411A>G	Coiled-coil Tail	38	Missense	VUS	Likely Pathogenic	10 y.o.	No	70–80	No

**Table 3:** ACMG Evidence Evaluation for VUS MYH9 Variants.

Variant (NM_002473.5)	Initial Classification	Final Classification	ACMG Evidence Codes Applied	Evidence Summary
**c.3838G>A**	VUS	Likely Pathogenic	PS4_Moderate, PM1, PM6, PP2	Reported in affected individuals; MYH9 hotspot; absent in population databases; phenotype consistent with MYH9-RD.
**c.3485+6C>T**	VUS	VUS (unchanged)	PM2_Supporting, BP4	Near splice donor; absent from controls; in silico effect uncertain; insufficient functional evidence.
**c.5411A>G**	VUS	Likely Pathogenic	PM1, PM2, PM6, PP2	Located in conserved functional domain; absent from controls; MYH9 intolerant to benign missense variation.

ACMG Criteria Key: PM1 = Located in mutational hotspot; PM2 = Absent from control populations; PM6 = Assumed de novo (without confirmed parental testing); PP2 = Missense variant in gene with low benign variation; PS4 = Increased prevalence in affected individuals; BP4 = Benign computational evidence.

## Data Availability

The data presented in this study are available on request from the corresponding author due to privacy and confidentiality restrictions pertaining to patient personal information.

## Abbreviations

The following abbreviations are used in this manuscript:

ITP	Immune thrombocytopenia
IT	Inherited thrombocytopenia
MYH9-RD	MYH9-related disease
VUS	Variant of unknown significance
TPO-RA	Trombopoetin receptor agonist
NGS	Next-generation sequencing
